# Bradycardia, Renal Failure, Atrioventricular Nodal Blockade, Shock, and Hyperkalemia (BRASH) Syndrome: A Frequently Overlooked and Underdiagnosed Condition

**DOI:** 10.7759/cureus.87601

**Published:** 2025-07-09

**Authors:** Malik Alqawasmi, Tomas Escobar Gil, Ketav Shah, Alexandra Millhuff, Nicholas Parchim

**Affiliations:** 1 Internal Medicine, University of New Mexico School of Medicine, Albuquerque, USA; 2 Emergency Medicine, University of New Mexico School of Medicine, Albuquerque, USA

**Keywords:** av nodal blockade, bradycardia, brash syndrome, hyperkalemia, renal failure, shock

## Abstract

Bradycardia, renal failure, atrioventricular nodal blockade, shock, and hyperkalemia (BRASH) syndrome is defined by the simultaneous presence of its five hallmark features. Despite its potentially severe clinical implications, it is frequently underdiagnosed due to symptom overlap with other conditions. Increased awareness and understanding of this syndrome are essential for prompt diagnosis and effective management, which are key to preventing serious morbidity. A 65-year-old male with a medical history of cirrhosis, portal hypertension, heart failure with reduced ejection fraction, chronic kidney disease, and ventricular bigeminy presented with dizziness and low blood pressure, which had started six to seven days prior. On initial examination, he was found to be bradycardic, with a heart rate in the 30s, and laboratory findings revealed hyperkalemia (6.6 mEq/L) and acute kidney injury (creatinine 2.9 mg/dL). The patient had been on a stable dose of metoprolol, an AV nodal blocking agent, which was recently reduced in the clinic due to worsening renal function. BRASH syndrome was suspected in the ED, prompting initiation of treatment with an epinephrine drip and insulin to address the hyperkalemia. Upon admission to the ICU, cardiac catheterization revealed nonobstructive coronary artery disease. The patient’s condition improved over five days, with normalization of potassium levels, restoration of normal sinus rhythm, and resolution of kidney injury, ultimately leading to recovery. At discharge, metoprolol was discontinued, and close cardiology follow-up was arranged. Recognizing BRASH syndrome as a multifaceted clinical entity is critical. Early identification and a comprehensive approach to managing the interdependent components - bradycardia, renal failure, and hyperkalemia - are essential to halt the syndrome’s progression and improve patient outcomes.

## Introduction

Bradycardia, renal failure, atrioventricular nodal blockade, shock, and hyperkalemia (BRASH) syndrome is a clinical constellation of signs and symptoms named after its five defining features. Although the relationship between atrioventricular (AV) nodal blocking agents and renal failure has long been recognized, this syndrome remains frequently underdiagnosed [1]. The recent adoption of the BRASH acronym serves as a helpful reminder for clinicians to maintain a high index of suspicion when encountering this specific combination of findings.

With a rapidly aging population and a growing burden of chronic disease, particularly among patients receiving goal-directed medical therapy for heart failure [2], understanding the proposed pathophysiology of BRASH syndrome is increasingly important. AV nodal blocking agents, such as beta-blockers and calcium channel blockers, can independently cause bradycardia. However, an inciting event, such as acute illness or the initiation of a new medication (e.g., an angiotensin-converting enzyme inhibitor), may trigger acute kidney injury (AKI), which in turn leads to hyperkalemia. Hyperkalemia further worsens bradycardia, especially in the presence of AV nodal blockade. This combination can result in profound bradycardia, decreased renal perfusion, and worsening hyperkalemia. If left unchecked, the resulting hypoperfusion can progress to multiorgan failure due to shock and worsening renal failure, thereby completing the BRASH pentad [3].

Importantly, patients typically present not due to the initial trigger, such as a new medication or dehydration-induced AKI, but because of symptoms resulting from the full manifestation of BRASH syndrome itself [1].

Distinguishing BRASH syndrome from isolated hyperkalemia is essential. While hyperkalemia alone can cause bradycardia and contribute to renal failure, bradycardia typically occurs only when serum potassium exceeds approximately 7 mEq/L. This threshold can aid in differentiating the two conditions. Additional clues that point to BRASH syndrome include the presence of an AV nodal blocking agent and an ECG lacking classic signs of hyperkalemia, such as hyperacute T waves [3]. Other helpful indicators include the absence of AV nodal blocker overdose, improvement with inotropic support, and resolution of arrhythmia following intravenous calcium administration [3].

Although often elusive, as the following case will illustrate, successful treatment of BRASH syndrome requires addressing all contributing components of the syndrome for effective recovery [4].

## Case presentation

The patient was a 65-year-old male with a known past medical history of cirrhosis with portal hypertension, heart failure with moderately reduced ejection fraction (EF) of 45-50%, chronic kidney disease, and ventricular bigeminy, who presented to the ED due to low blood pressure readings at home and subjective symptoms of dizziness and lightheadedness experienced that morning.

During his most recent cardiology clinic visit, approximately one month earlier, he was noted to have hyperkalemia. At that time, his metoprolol and furosemide doses were reduced in the context of worsening renal function, and spironolactone was discontinued due to hyperkalemia.

In the ED, the patient was found to be bradycardic, with a heart rate in the 30s beats per minute, and hypotensive, with a systolic blood pressure ranging from 80 to 90 mmHg. Laboratory findings revealed a serum potassium level of 6.6 mEq/L, a creatinine of 2.9 mg/dL (baseline approximately 2.5 mg/dL), and a B-type natriuretic peptide level of 14,453 pg/mL (Table 1). A chest X-ray showed mild bilateral pulmonary edema. Physical examination revealed jugular venous distension and 2+ pitting edema in the bilateral lower extremities. Hypervolemia was confirmed on bedside echocardiography, which demonstrated a distended inferior vena cava and a dilated right ventricle.

**Table 1:** Key laboratory findings on presentation with reference ranges Laboratory values obtained at presentation in a patient with BRASH syndrome demonstrate hyperkalemia, AKI, and elevated natriuretic peptide levels. Reference ranges represent standard adult values and may vary slightly depending on the laboratory. AKI, acute kidney injury; BRASH, bradycardia, renal failure, atrioventricular nodal blockade, shock, and hyperkalemia

Laboratory test	Patient value	Reference range
Serum potassium	6.6 mEq/L	3.5-5.1 mEq/L
Serum creatinine	2.9 mg/dL	0.6-1.3 mg/dL (varies by age and sex)
Baseline creatinine	2.5 mg/dL	0.6-1.3 mg/dL (varies by age and sex)
B-type natriuretic peptide	14,453 pg/mL	<100 pg/mL

Given the patient’s hypotension, hyperkalemia, and home use of the AV nodal blocking agent metoprolol, there was initial suspicion for BRASH syndrome in the ED. He was started on an epinephrine drip and received insulin in accordance with the hospital’s hyperkalemia protocol.

**Figure 1:**
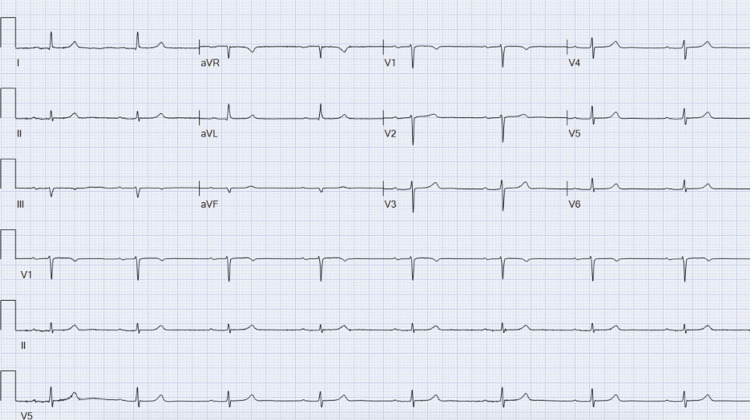
ECG showing first-degree AV block with sinus bradycardia at a ventricular rate of 49 beats per minute AV, atrioventricular

**Figure 2:**
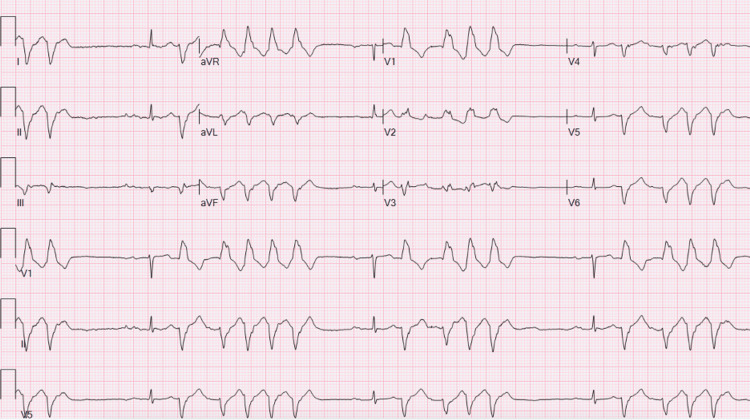
ECG demonstrating first-degree AV block with frequent PVCs and no evidence of ischemic changes AV, atrioventricular; PVC, premature ventricular contraction

Cardiology was consulted, and upon reviewing the patient’s ECGs, they noted bradycardia (Figure 1), first-degree AV block (Figure 1, Figure 2), frequent premature ventricular contractions (PVCs) (Figure 2), and no ischemic changes (Figure 1, Figure 2). Notably, hyperacute T-waves were absent. The cardiology team concurred with the preliminary diagnosis of BRASH syndrome and recommended holding his cardiac medications - particularly metoprolol - monitoring renal function closely, and continuing treatment for hyperkalemia.

After initial stabilization in the ED and transfer to the ICU, the patient underwent a diagnostic coronary angiogram in the catheterization lab to rule out ischemic disease as the underlying cause of his cardiomyopathy. The angiogram revealed nonobstructive coronary artery disease, and aspirin and statin therapy were recommended for secondary prevention. However, these findings did not fully account for the patient’s clinical presentation, and the cardiology team advised further evaluation for nonischemic etiologies potentially contributing to BRASH syndrome.

The patient recovered after a five-day stay in the medical ICU. Management during this period focused on addressing the core components of BRASH syndrome, primarily treating hyperkalemia with insulin and managing the shock state with epinephrine. His AKI resolved, with serum creatinine returning to baseline, and his potassium levels normalized. He was subsequently transferred to the cardiac unit, with plans for a scheduled ablation procedure to address his frequent PVCs. These ectopic beats were presumed to contribute to his reduced EF, although this remained a hypothesis pending longitudinal assessment of his cardiac function.

## Discussion

**Figure 3:**
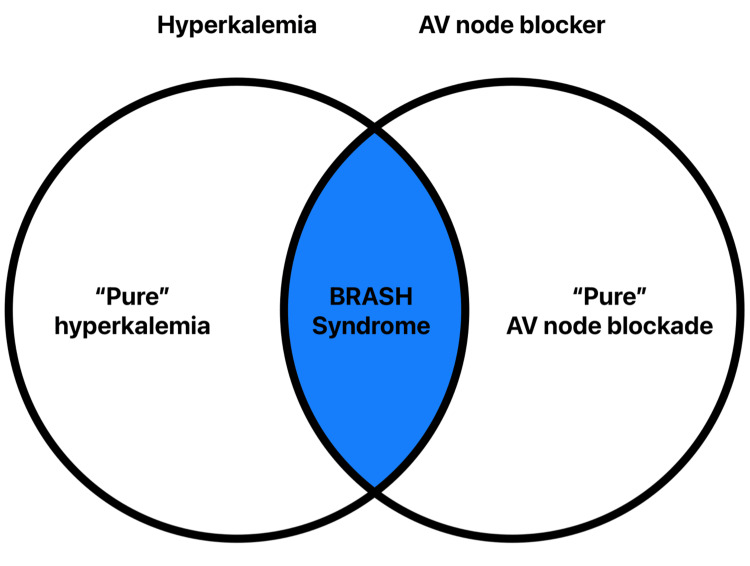
BRASH syndrome arises at the intersection of hyperkalemia and AV nodal blockade AV, atrioventricular; BRASH, bradycardia, renal failure, atrioventricular nodal blockade, shock, and hyperkalemia Adapted from Farkas et al. [1] and Majeed et al. [4]; original figure created by the authors for this manuscript based on conceptual inspiration. No copyrighted material was reused.

Management of patients with BRASH syndrome often occurs without recognition of the interconnected nature of its individual components [1]. While standard treatments, such as discontinuing the AV nodal blocking agent, can be effective when addressing isolated manifestations, early identification of the syndrome allows for a more integrated treatment strategy and more rapid recovery. Hyperkalemia is believed to play a synergistic role in precipitating bradycardia (Figure 3), making its prompt and aggressive management a cornerstone of therapy [4]. Additionally, timely outpatient follow-up - ideally within seven days of hospital discharge - is crucial to prevent recurrence and avoid reentering the self-perpetuating cycle that characterizes BRASH syndrome.

**Figure 4:**
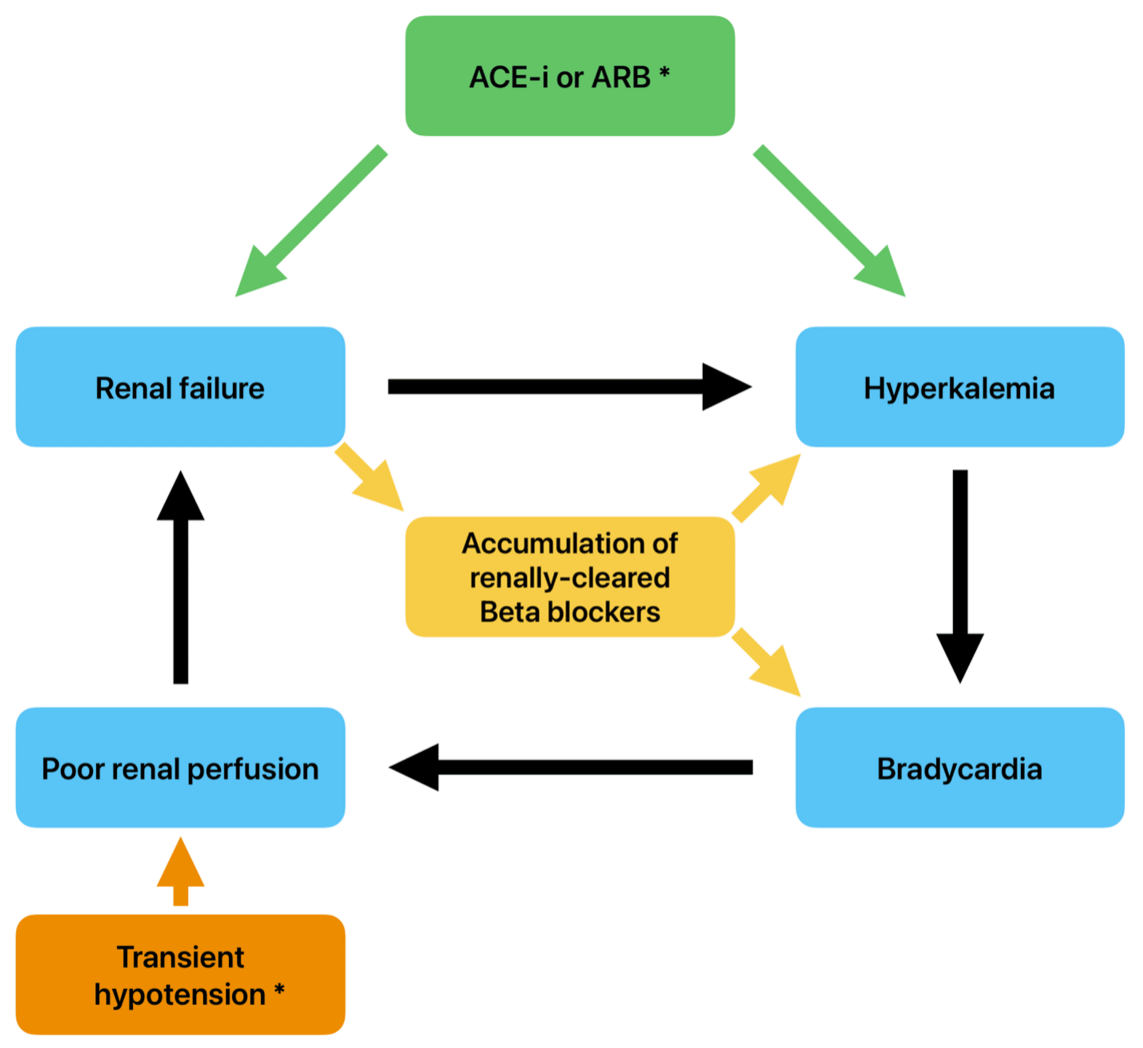
Pathophysiology of BRASH syndrome ^*^ Indicates contributing but nonessential factors ACE-i, angiotensin-converting enzyme inhibitor; ARB, angiotensin-receptor blocker; BRASH, bradycardia, renal failure, atrioventricular nodal blockade, shock, and hyperkalemia Adapted from Farkas et al. [1]; original figure created by the authors for this manuscript based on conceptual inspiration. No copyrighted material was reused.

It is also important to distinguish BRASH syndrome from isolated AV nodal blocker toxicity, given its intertwined pathophysiology involving hyperkalemia and renal hypoperfusion. The condition can be further exacerbated when the AV nodal blocking agent is primarily renally excreted [1]. A common pitfall in managing BRASH is focusing exclusively on one component, such as discontinuing the AV nodal blocker, without addressing the other contributing factors (Figure 4) [1].

It is important to emphasize that the patient received epinephrine in the ED, which likely contributed to his recovery and favorable outcome. Although the American Heart Association’s Advanced Cardiovascular Life Support (ACLS) guidelines recommend atropine for patients with symptomatic bradycardia [5], this approach is not suitable for BRASH syndrome, as the bradycardia is not vagally mediated. Instead, the preferred treatment for severe bradycardia in BRASH is epinephrine or isoproterenol [6].

Most cases of BRASH syndrome are mild and respond well to standard medical interventions [3]. A key clinical consideration is whether AV nodal blocking agents should be reintroduced, particularly since these medications are foundational in the management of heart failure [7]. Once hyperkalemia and AKI have resolved - indicated by normalization of serum potassium and return of serum creatinine to baseline - it is generally considered safe to restart beta-blockers or calcium channel blockers.

Equally important is educating patients about the constellation of symptoms associated with arrhythmias and BRASH syndrome. Patients should be advised to seek prompt medical attention if they experience signs such as syncope or near-syncope, fatigue, or dyspnea. In more severe cases, dialysis or transvenous pacing may be required as temporizing measures to interrupt the cycle [1]. However, a comprehensive and holistic approach targeting all components of the syndrome is essential for effective long-term management.

## Conclusions

BRASH syndrome represents a self-perpetuating cycle involving bradycardia, renal failure, AV nodal blockade, shock, and hyperkalemia. While the ED management of each component is grounded in established treatments, early recognition of the syndrome enables a more integrated and effective therapeutic approach. Unlike standard ACLS protocols, BRASH management requires epinephrine instead of atropine. Therefore, timely identification and appropriate intervention are critical to halting and reversing the progression to shock. Given the potential for recurrence, it is essential that frontline clinicians become familiar with BRASH syndrome and incorporate its recognition and management into their clinical protocols.

## References

[1] Farkas JD, Long B, Koyfman A, Menson K (2020). BRASH syndrome: bradycardia, renal failure, av blockade, shock, and hyperkalemia. J Emerg Med.

[2] Beard JR, Officer A, de Carvalho IA (2016). The World report on ageing and health: a policy framework for healthy ageing. Lancet.

[3] Lizyness K, Dewald O (2025). BRASH syndrome. StatPearls [Internet].

[4] Majeed H, Khan U, Khan AM (2023). BRASH syndrome: a systematic review of reported cases. Curr Probl Cardiol.

[5] Panchal AR, Bartos JA, Cabañas JG (2020). Part 3: Adult Basic and Advanced Life Support: 2020 American Heart Association Guidelines for cardiopulmonary resuscitation and emergency cardiovascular care. Circulation.

[6] Shah P, Silangruz K, Lee E, Nishimura Y (2022). Two cases of BRASH syndrome: a diagnostic challenge. Eur J Case Rep Intern Med.

[7] Metra M, Teerlink JR (2017). Heart failure. Lancet.

